# A rare case of extramedullary hematopoiesis: when the pericardium produces blood cells

**DOI:** 10.1186/s43044-025-00680-w

**Published:** 2025-09-11

**Authors:** SALAH-EDDINE HAYAR, MEHDI TAMIR, MOHAMED KHALDI, MAHA BOUZIANE, MERYEM HABOUB, SALIM AROUS, MOHAMED GHALI BENNOUNA, ABDENASSER DRIGHIL, RACHIDA HABBAL, ABDERRAHMANE MELLOUKI

**Affiliations:** 1 Cardiology Department, Ibn Rochd University Hospital Center, Casablanca, Morocco; 2 Pathology Department, Ibn Rochd University Hospital Center, Casablanca, Morocco

**Keywords:** Case report, Polycythemia vera, Myelofibrosis, Hematopoiesis, Tamponade

## Abstract

**Background:**

Cardiac tamponade is an extreme cardiological emergency, fatal in the absence of rapid intervention. This case report highlights a noteworthy and rare correlation between post-polycythemia vera myelofibrosis and extramedullary hematopoiesis affecting the pericardium, leading to tamponade or pericardial effusion.

**Case presentation:**

A 69-year-old female with a history of polycythemia vera presented with worsening dyspnea, fever, and altered condition. Examination revealed low blood pressure, tachycardia, jugular vein distention, and muffled heart sounds, leading to a diagnosis of cardiac tamponade due to a large pericardial effusion. Emergency pericardiocentesis was performed, revealing serosanguineous fluid with signs of clonally proliferative hematopoietic cells, indicating possible progression to myelofibrosis. Bone marrow biopsy confirmed post-polycythemia vera myelofibrosis. The patient’s condition improved, and she was referred back to her hematologist for further management.

**Conclusion:**

Increased awareness may improve early diagnosis and treatment, ultimately enhancing patient outcomes.

**Supplementary Information:**

The online version contains supplementary material available at 10.1186/s43044-025-00680-w.

## Background

Cardiac tamponade is an extreme cardiological emergency, fatal in the absence of rapid intervention. Etiological diagnosis remains a real challenge, conditioning subsequent management (1, 2). This case report highlights a noteworthy correlation between post-polycythemia vera myelofibrosis (PPV-MF) and extramedullary hematopoiesis (EMH) affecting the pericardium, leading to tamponade or pericardial effusion.

## Case presentation

This is the case of a 69-year-old female hypertensive patient, treated since 2019 for polycythemia vera with positive JAK 2 mutation, who presented to the emergency department with progressively worsening dyspnea, fever and altered general condition characterized by asthenia, easy fatigability, and unquantified weight loss.

Examination revealed a blood pressure of 120/70 mmHg, tachycardia at 104 bpm with jugular veins distention, and muffled heart sounds. The EKG showed sinus tachycardia, and transthoracic echocardiography revealed a large pericardial effusion with signs of cardiac tamponade.

An emergency pericardiocentesis was performed, with a good clinical outcome. The pericardial fluid was serosanguineous, with a protein level of 46 g/l, cytology examination revealed immature erythroid and granulocytic cells, suggestive of clonally proliferative hematopoietic cells. Adenosine deaminase levels in pericardial fluid were normal and sputum tests for Mycobacterium tuberculosis were negative. Complete blood count showed an elevated leukocyte count of 22 × 10^9 cells/L, hemoglobin of 10 g/dl, and platelet count of 1,158,000/L. Extramedullary hematopoiesis (EMH) with concomitant anemia was suspected as evolution of his polycythemia vera to myelofibrosis.

The patient subsequently underwent a bone marrow biopsy with pathology consistent with post-polycythemia vera myelofibrosis.

The evolution was marked by a complete regression of the effusion and the patient was referred back to her hematologist for further management.

## Discussion

Polycythemia vera, also known as Vaquez disease, is a myeloproliferative neoplasm characterized by the overproduction of red blood cells due to a mutation in the JAK2 gene. This disorder often leads to increased blood viscosity and a higher risk of thrombotic events. As the disease progresses, patients may develop myelofibrosis, a condition marked by replacing bone marrow with fibrous tissue, resulting in ineffective hematopoiesis and subsequent cytopenias (3). In advanced stages, extramedullary hematopoiesis can occur as the body attempts to compensate for inadequate blood cell production in the marrow, leading to hematopoietic activity in atypical sites, such as the spleen and, in rare cases, the pericardium (4–6).

The development of cardiac tamponade due to extramedullary hematopoiesis (EMH) in the pericardium is a rare but significant complication(7). EMH can lead to the accumulation of hematopoietic tissue, resulting in increased fluid production and subsequent pericardial effusion.

This condition has been reported very rarely in the literature, with around a dozen case reports published (7, 8).

Patients may present with nonspecific symptoms such as dyspnea, fatigue, or signs of heart failure. Clinical evaluation often reveals tachycardia and hypotension, indicative of impaired cardiac filling due to fluid accumulation. Diagnosis typically involves echocardiography, which can confirm the presence of pericardial effusion and assess hemodynamic compromise (1).

**Fig. 1:**
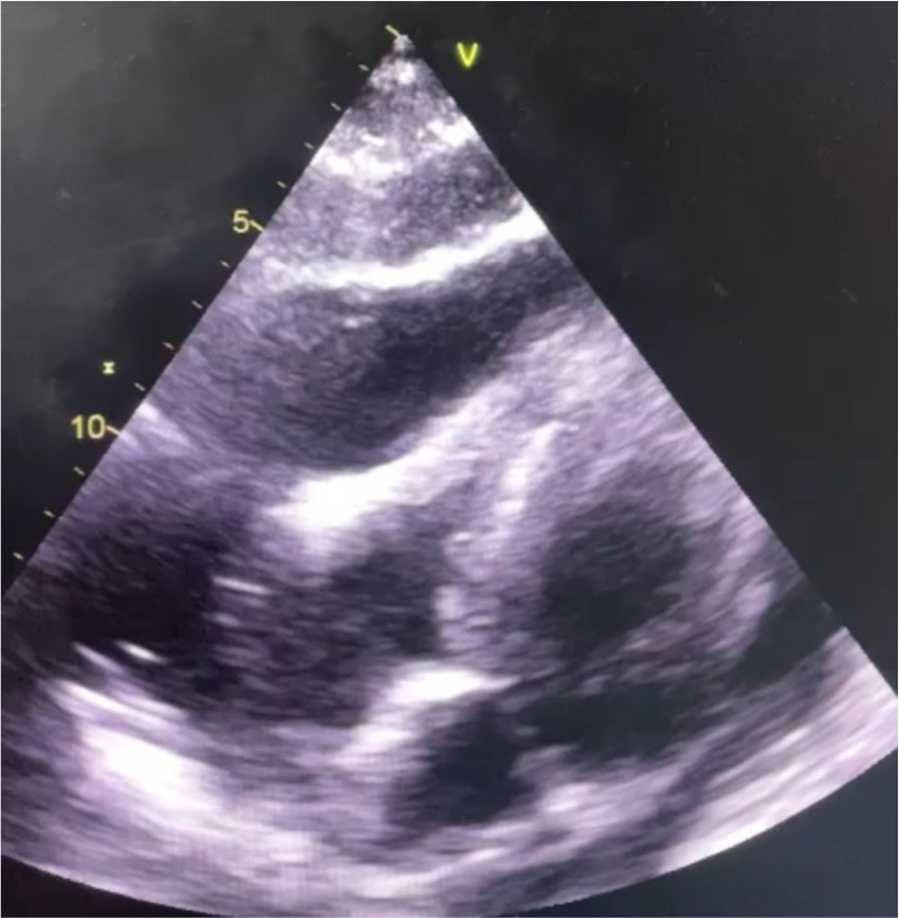
TTE showing large pericardial effusion with RV collapse

**Fig. 2:**
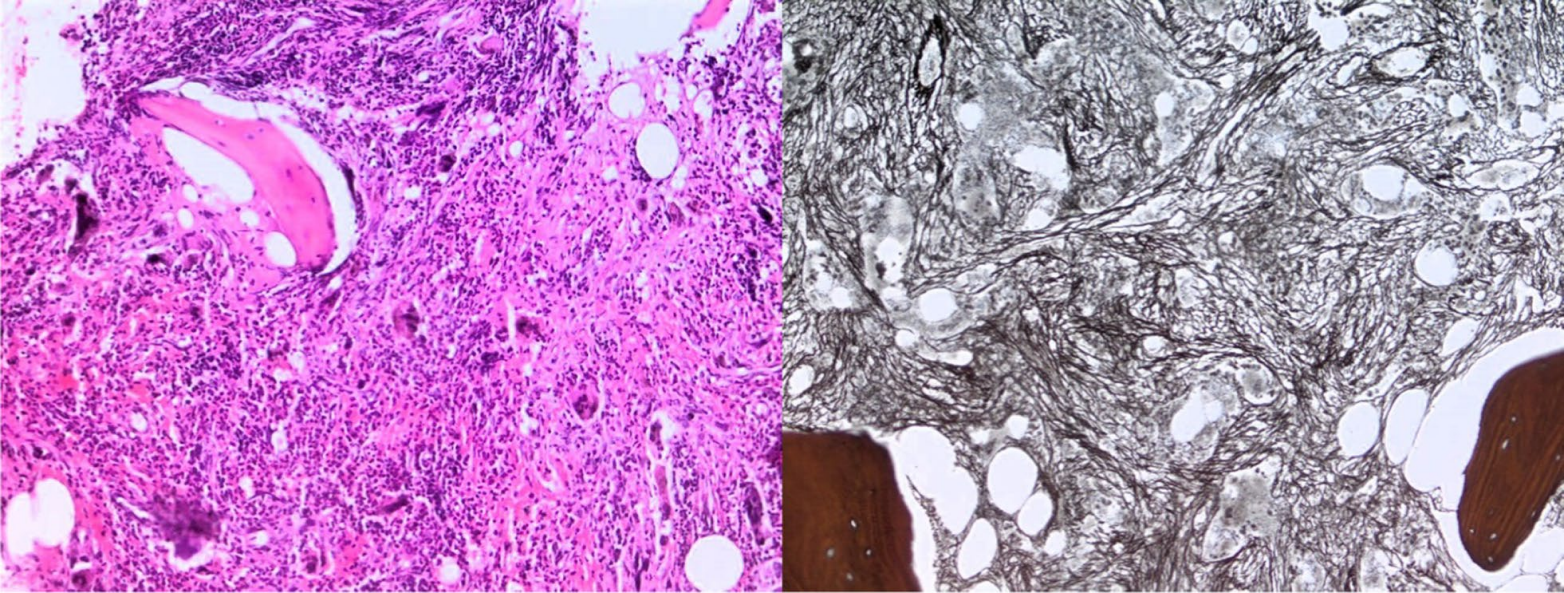
Bone marrow biopsy showing extensive myelofibrosis

Differential diagnoses for cardiac tamponade and pericardial effusion include conditions such as infectious pericarditis, malignancy-related effusions, and postsurgical changes (1, 7). To confirm a diagnosis of extramedullary hematopoiesis (EMH), a thorough evaluation is necessary. This typically includes cytological analysis of the pericardial fluid, which may reveal immature hematopoietic cells, and imaging studies such as MRI or CT to identify any ectopic hematopoietic tissue (6, 9). Additionally, a bone marrow biopsy may be performed to assess for underlying myelofibrosis or other hematological disorders that could contribute to the EMH (7). Integrating these findings allows for a comprehensive diagnosis and guides appropriate management strategies (Figs. 1, 2).

In our patient, the history of polycythemia vera prompted us to consider this diagnosis, leading us to perform a bone marrow biopsy that revealed significant myelofibrosis.

The management generally necessitates a dual approach: immediate intervention for the effusion and long-term treatment of the underlying hematologic condition.

A review of the literature revealed nine case reports of cardiac tamponade associated with myelofibrosis. Treatment was documented for seven patients, with two undergoing pericardial fenestration, one receiving both pericardial fenestration and pericardiocentesis, and two patients treated with pericardiocentesis and radiotherapy. Additionally, one patient was managed with a combination of pericardial fenestration, pericardiocentesis, and radiotherapy. Complete resolution of symptoms was observed in those who underwent pericardial fenestration. Unfortunately, one patient who received both pericardial fenestration and pericardiocentesis was reported to have died. Among the two patients treated with the combination of pericardial fenestration, pericardiocentesis, and radiotherapy, both achieved complete resolution, while one patient experienced only partial resolution(8).

Our patient was monitored for one month after the pericardiocentesis, with no recurrence noted. She was then transferred to the hematology department for further evaluation of her underlying condition and appropriate treatment.

## Conclusion

In conclusion, although cardiac tamponade resulting from extramedullary hematopoiesis (EMH) in patients with post-polycythemia vera myelofibrosis (PPV-MF) is uncommon, its consequences can be significant. Heightened awareness of this complication can lead to earlier diagnosis and intervention, thereby enhancing patient outcomes. Future research should aim to clarify the pathophysiological mechanisms that increase the risk of EMH and investigate new therapeutic strategies to address these challenges.

## Supplementary Information

Additional file1 (DOCX 81 KB)

## Data Availability

No datasets were generated or analysed during the current study.

## Abbreviations

PPV-MF: Post-polycythemia vera myelofibrosis.
EMH: Extramedullary hematopoiesis.
MRI: Magnetic resonance imaging.
CT: Computed tomography
