# Supplementary material for: A rare case of extramedullary hematopoiesis: when the pericardium produces blood cells
Source: Egypt Heart J. 2025 Sep 11;77:86. doi: 10.1186/s43044-025-00680-w (PMC12425877; doi:10.1186/s43044-025-00680-w)
Supplement: Supplementary file 1 — Additional file1 (DOCX 81 KB) [file 43044_2025_680_MOESM1_ESM.docx]

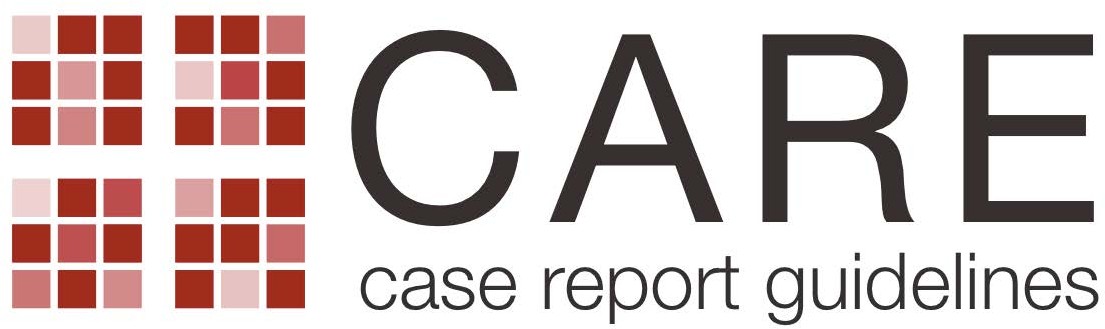
CARE Checklist of information to include when writing a case report
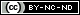


**Topic Item Checklist item description Reported on Line**

**Title 1** The diagnosis or intervention of primary focus followed by the words “case report” YES

**Key Words 2** 2 to 5 key words that identify diagnoses or interventions in this case report, including "case report" YES

**Abstract**

**(no references)**

**3a** Introduction: What is unique about this case and what does it add to the scientific literature? YES

**3b** Main symptoms and/or important clinical findings YES

**3c** The main diagnoses, therapeutic interventions, and outcomes YES

**3d** Conclusion—What is the main “take-away” lesson(s) from this case? YES

**Introduction 4** One or two paragraphs summarizing why this case is unique (**may include** reference**s**) BACKGROUND

**Patient Information 5a** De-identified patient specific information CASE PRESENTATON

**5b** Primary concerns and symptoms of the patient YES

**5c** Medical, family, and psycho-social history including relevant genetic information YES

**5d** Relevant past interventions with outcomes YES

**Clinical Findings**

**Timeline**

**Diagnostic Assessment**

**Therapeutic Intervention**

**Follow-up and Outcomes**

1. Describe significant physical examination (PE) and important clinical findings CASE PRESENTATION
2. Historical and current information from this episode of care organized as a timeline CASE PRESENTATON

**8a** Diagnostic testing (such as PE, laboratory testing, imaging, surveys). CASE PRESENTATON

**8b** Diagnostic challenges (such as access to testing, financial, or cultural) N/A

**8c** Diagnosis (including other diagnoses considered) YES

**8d** Prognosis (such as staging in oncology) where applicable N/A

**9a** Types of therapeutic intervention (such as pharmacologic, surgical, preventive, self-care) CASE PRESENTATON

**9b** Administration of therapeutic intervention (such as dosage, strength, duration) YES

**9c** Changes in therapeutic intervention (with rationale) N/A

**10a** Clinician and patient-assessed outcomes (if available) CASE PRESENTATON

**10b** Important follow-up diagnostic and other test results YES

**10c** Intervention adherence and tolerability (How was this assessed?) N/A

**10d** Adverse and unanticipated events N/A

**Discussion 11a** A scientific discussion of the strengths AND limitations associated with this case report DISCUSSION

**11b** Discussion of the relevant medical literature **with references** YES

**11c** The scientific rationale for any conclusions (including assessment of possible causes) YES

**11d** The primary “take-away” lessons of this case report (without references) in a one paragraph conclusion YES

**Patient Perspective 12** The patient should share their perspective in one to two paragraphs on the treatment(s) they received N/A

**Informed Consent 13** Did the patient give informed consent? Please provide if requested . . . . . . . . . . . . . . . . . . . . . . . . . . . . . . . . . . . . . . **Yes X No**
